# Calcifying Bowel Inflammation: A Case Report

**DOI:** 10.1155/2010/526486

**Published:** 2010-05-17

**Authors:** Jacques Klein, Philippe Morel, Christian Toso

**Affiliations:** ^1^Abdominal Surgery, Department of Surgery, University Hospitals of Geneva, rue Gabrielle-Perret-Gentil 4, 1211 Genève 14, Switzerland; ^2^Abdominal and Transplant Surgery, Department of Surgery, University Hospitals of Geneva, rue Gabrielle-Perret-Gentil 4, 1211 Genève 14, Switzerland

## Abstract

We report about a previously healthy 72-year-old woman, presented with 6 days of left lower quadrant abdominal pain and constipation. There was no report of fever, melena, hematochezia or change in appetite. The physical exam demonstrated a distended abdomen with palpable left lower quadrant pain, without guarding. CT showed images compatible with a sigmoid diverticulitis and a calcification of the sigmoid colon. After antibiotic threatment, a colonoscopy was performed which revealed the presence of a shell in the sigmoid colon. Our case illustrates the need for a colonoscopy following an attack of diverticulitis to look for a cancer or rarely a foreign body.

A previously healthy 72-year-old woman presented with 6 days of left lower quadrant abdominal pain and constipation. There were no report of fever, melena, hematochezia or change in appetite. The physical exam demonstrated a distended abdomen with palpable left lower quadrant pain, without guarding. The blood work revealed a normal white blood cell count but an increased C-reactive protein (110 mg/L). 

CT showed images compatible with a sigmoid diverticulitis and a calcification of the sigmoid colon (Figure 1(a), arrow). 

The patient was treated with IV antibiotics (Ceftriaxone 2 g daily and Metronidazole 500 mg three times daily), with an adequate resolution of her signs and symptoms.

A colonoscopy performed one week later revealed the presence of a shell in the sigmoid colon (at 25 cm from the anus), which was extracted without complication (Figures 1(b) and 1(c)). The patient subsequently confessed to have eaten shellfish in paella the week prior to admission. 

Diverticulosis is a common illness in developed countries where it is present in 50% of people over the age of 60. The sigmoid colon is the most commonly affected. Approximately 10% of patients, who present with diverticulosis, will develop diverticulitis, usually caused by a fecalith. 

The literature reports a few cases of diverticulitis caused by the presence of a foreign body (generally chicken or fish bones) [1, 2]. In 88% of cases the patients are unconscious about the ingested foreign body; furthermore, in 76% of cases the foreign body passes spontaneously without complication [2, 3]. Though rarely, the actual incidence of diverticulitis caused by a foreign body is unknown. The predisposing risk factor for ingestion is the use of dentures which reduces the sensibility of the palate (72%); however, this was not the case for our patient [2]. About 10%–20% of all ingested foreign bodies must be removed endoscopically and 1%–14% need operative removal [3].

Our case illustrates the need for a colonoscopy following an attack of diverticulitis to look for a cancer or rarely a foreign body. The patient left hospital after completing antibiotic treatment without complication. She remained asymptomatic three months after discharge.

## Figures and Tables

**Figure 1:**
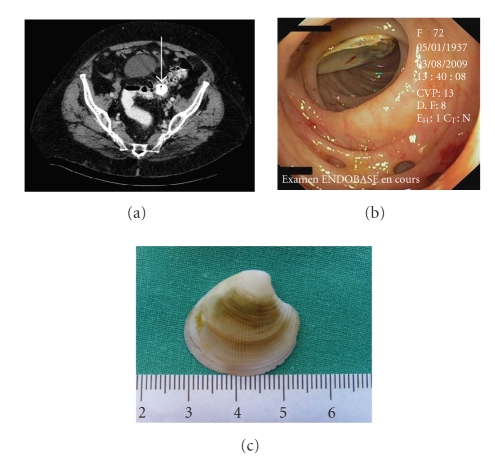


## References

[1] Lubel J, Wiley M (2005). Images of interest: foreign bodies and diverticulitis. *Journal of Gastroenterology and Hepatology*.

[2] Rodríguez-Hermosa JI, Codina-Cazador A, Sirvent JM, Martín A, Gironès J, Garsot E (2008). Surgically treated perforations of the gastrointestinal tract caused by ingested foreign bodies. *Colorectal Disease*.

[3] Velitchkov NG, Grigorov GI, Losanoff JE, Kjossev KT (1996). Ingested foreign bodies of the gastrointersintal tract: retrospective analysis of 542 cases. *World Journal of Surgery*.

